# SHARED SOCIAL ACCOUNT: UNDERSTANDING INTERGENERATIONAL COLLABORATION AND INTERACTION ON XIAOHONGSHU

**DOI:** 10.1093/geroni/igae098.2733

**Published:** 2024-12-31

**Authors:** Tongxin Sun, Tongtong Jin, Qiong Zhang, Yuanyuan Liu

**Affiliations:** Tsinghua University, Beijing, Beijing, China (People’s Republic); Tsinghua University, Beijing, Beijing, China (People’s Republic); School of Mechanical Engineering & Automation, Beijing, Beijing, China (People’s Republic); School of Mechanical Engineering & Automation, Beihang University, Beijing, Beijing, China (People’s Republic)

## Abstract

Xiaohongshu, a social media platform in China known for lifestyle sharing through text, images, and videos, is predominantly popular among the younger generation. It has seen a remarkable uptick in older adult users in the post-pandemic era, with a notable phenomenon in “shared accounts” that feature content co-created by grandparent-grandchild pairs. These accounts have garnered significant attention for their unique intergenerational content and elicited emotional resonance within young audiences. Our study utilized a mixed-methods approach, including web scraping and interviews, to investigate the motivations and experiences underpinning the content creation process on these shared accounts. Through cluster analysis of content topics and in-depth interviews with four grandparent-grandchild pairs actively managing shared accounts, we explored their creative collaboration process and the influence of digital technology. Our research uncovered a symbiotic exchange of knowledge between generations: grandparents shared their life wisdom and experiences, while grandchildren introduced them to digital platforms and current social trends, enabling the transformation of shared narratives into media forms accessible to a wider audience. This collaborative creation process not only enhanced emotional communication within families but also fostered intergenerational social interactions externally. However, grandparents, due to their lack of perceived confidence in digital literacy, typically tended to pass control to their grandchildren for the restructuring of narratives, leading to technological disparities within the shared account. This study sheds light on the influence of technology on collaborative narratives across generations and discusses ways to advance technological equity to empower older adults in achieving self-expression and meaningful social engagement.

